# HIV-1 subtype C Nef-mediated SERINC5 down-regulation significantly contributes to overall Nef activity

**DOI:** 10.1186/s12977-023-00618-7

**Published:** 2023-03-31

**Authors:** Delon Naicker, Nelson Sonela, Steven W. Jin, Takalani Mulaudzi, Doty Ojwach, Tarylee Reddy, Mark A. Brockman, Zabrina L. Brumme, Thumbi Ndung’u, Jaclyn K. Mann

**Affiliations:** 1 HIV Pathogenesis Programme, The Doris Duke Medical Research Institute, University of KwaZulu-Natal, Durban, 4001 South Africa; 2 Chantal BIYA International Reference Centre for Research on HIV/AIDS Prevention and Management (CIRCB), P.O. Box 3077, Yaoundé, Cameroon; 3 Faculty of Health Sciences, Simon Fraser University, Burnaby, BC V5A 1S6 Canada; 4 Medical Research Council, Biostatistics Unit, Durban, 4001 South Africa; 5 Molecular Biology and Biochemistry, Simon Fraser University, Burnaby, BC V5A 1S6 Canada; 6 British Columbia Centre for Excellence in HIV/AIDS, Vancouver, BC V6Z 1Y6 Canada; 7 Africa Health Research Institute, Durban, 4001 South Africa; 8 Ragon Institute of Massachusetts General Hospital, Massachusetts Institute of Technology and Harvard University, Cambridge, MA USA; 9 Division of Infection and Immunity, University College London, London, WC1E 6BT UK

**Keywords:** HIV-1 Nef, HIV-1 subtype C, SERINC5 down-regulation

## Abstract

**Background:**

Nef performs multiple cellular activities that enhance HIV-1 pathogenesis. The role of Nef-mediated down-regulation of the host restriction factor SERINC5 in HIV-1 pathogenesis is not well-defined. We aimed to investigate if SERINC5 down-regulation activity contributes to HIV-1 subtype C disease progression, to assess the relative contribution of this activity to overall Nef function, and to identify amino acids required for optimal activity. We measured the SERINC5 down-regulation activity of 106 subtype C Nef clones, isolated from individuals in early infection, for which the Nef activities of CD4 and HLA-I down-regulation as well as alteration of TCR signalling were previously measured. The relationship between SERINC5 down-regulation and markers of disease progression, and the relative contribution of SERINC5 down-regulation to a Nef fitness model-derived E value (a proxy for overall Nef fitness in vivo), were assessed.

**Results:**

No overall relationship was found between SERINC5 down-regulation and viral load set point (*p* = 0.28) or rate of CD4^+^ T cell decline (*p* = 0.45). CD4 down-regulation (*p* = 0.02) and SERINC5 down-regulation (*p* = 0.003) were significant determinants of E values in univariate analyses, with the greatest relative contribution for SERINC5 down-regulation, and only SERINC5 down-regulation remained significant in the multivariate analysis (*p* = 0.003). Using a codon-by-codon analysis, several amino acids were significantly associated with increased (10I, 11V, 38D, 51T, 65D, 101V, 188H and, 191H) or decreased (10K, 38E, 65E, 135F, 173T, 176T and, 191R) SERINC5 down-regulation activity. Site-directed mutagenesis experiments of selected mutants confirmed a substantial reduction in SERINC5 down-regulation activity associated with the mutation 173T, while mutations 10K, 135F, and 176T were associated with more modest reductions in activity that were not statistically significant.

**Conclusions:**

These results suggest that SERINC5 down-regulation is a significant contributor to overall Nef function and identify potential genetic determinants of this Nef function that may have relevance for vaccines or therapeutics.

**Supplementary Information:**

The online version contains supplementary material available at 10.1186/s12977-023-00618-7.

## Introduction

HIV-1 Nef is a small accessory protein that optimises the cellular environment for viral replication and viral immune evasion through internalising various receptors, including CD4, human leukocyte antigen-I (HLA-I), and serine incorporator (SERINC) proteins [1], and modulating cell signalling activities [2]. The relative importance of these various Nef activities for HIV disease progression is not completely known. Previous studies using animal models indicated that Nef-mediated CD4 down-regulation and enhancement of infectivity are likely major contributors to Nef-mediated enhancement of pathogenicity [3, 4]. In natural subtype B infection, elite controllers have impairment in multiple Nef functions [5, 6], while in progressors only Nef-driven virion infectivity has been associated with markers of disease progression [7]. These studies suggest that Nef-driven infectivity significantly influences pathogenesis, however the extent to which natural variation in Nef-driven infectivity, or the drivers thereof, influence clinical outcome in HIV-1 subtype C infection remains unknown.

In recent years, Nef-mediated SERINC down-regulation from the cell surface was found to be an important mechanism by which Nef enhances viral infectivity [8, 9]. The incorporation of SERINC proteins (specifically SERINC3 and SERINC5) into virions impairs virion cell entry, and this antiviral activity is more potent for SERINC5 than SERINC3. Several Nef motifs required for CD4 down-regulation are also required for SERINC5 down-regulation, including CAW (57–59), RR (105–106), LL (164–165), and E/DD (174–175) [10, 11]. In addition, Nef residues G2, I109, L112, W115, and F121 are required for the interaction between Nef and SERINC5 [10]. These residues are seldom mutated in natural sequences however, and while the effect of natural Nef polymorphisms on SERINC5 down-regulation ability has been studied in subtype B [6, 12, 13], there are fewer studies investigating the effect of natural variation in subtype C on this Nef function [14]. The identification of amino acid variants required for optimal Nef function could reveal antiviral targets.

Given the key role of Nef-mediated SERINC5 down-regulation in enhancing virion infectivity, we sought to determine whether this Nef activity contributes significantly to HIV-1 subtype C disease progression. The SERINC5 down-regulation activity of 106 Nef clones, isolated from patients in early infection and for which CD4 and HLA-I down-regulation activities [15] as well as alteration of TCR signalling activity [16] had previously been measured, was evaluated in a CEM-derived CD4 + T cell line using a flow cytometry-based assay and then related to subsequent rate of CD4 + T cell decline and viral load set point. In past work, computational models of the Nef fitness landscape that were based on correlated patterns of multiple mutations and generated using > 10 000 Nef sequences, were used to generate a predicted Nef fitness measure—an E value—for each of the same Nef clones [17]. Since the model E values were able to predict individual Nef functions measured in vitro (although not with as much accuracy as for similar models of the conserved Gag protein), E values were assumed to be a reasonable proxy for overall Nef function in vivo, which is a composite of the multiple Nef functions [17]. Here, we used the E values of the patient-derived Nef clones to investigate the individual contribution of each of the Nef functions, including SERINC5 down-regulation, to overall Nef function. While SERINC5 down-regulation did not directly associate with markers of disease progression overall, SERINC5 down-regulation and CD4 down-regulation were both significantly associated with the Nef fitness model-derived E values, suggesting that these Nef functions may have a higher contribution to overall Nef function in vivo than the other Nef functions studied here. Further, we identified natural variation in subtype C Nef that is associated with altered SERINC5 down-regulation ability.

## Results

### SERINC5 down-regulation activity correlates with CD4 down-regulation activity

**Fig. 1:**
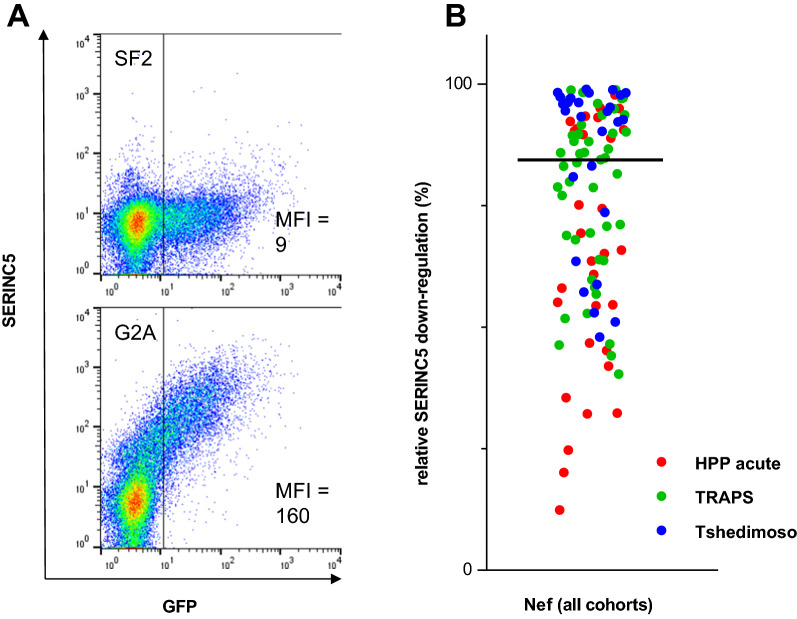
SERINC5 down-regulation activities of patient-derived Nef clones. **A** Representative flow cytometry plots of the positive control (SF2 Nef) and negative control (SF2 Nef with the G2A mutation, rendering it inactive for SERINC5 down-regulation) are shown. The median fluorescence intensity (MFI) values indicate SERINC5 cell-surface expression in green fluorescent protein (GFP) expressing cells (representing cells transfected with Nef clones). **B** The SERINC5 down-regulation activities of Nef clones derived from patients from the HPP acute infection, TRAPS and Tshedimoso cohorts are shown. SERINC5 down-regulation activities of Nef clones were expressed relative to SF2 (100% down-regulation) and G2A (0% down-regulation)

The Nef-mediated SERINC5 down-regulation ability was determined for 106 Nef clones, each isolated from a unique individual in early/acute HIV-1 subtype C infection, to assess the effect of this Nef function on subsequent disease progression as well as its contribution to overall Nef function. The Nef clones used for this study were prepared in a previous study [15], which confirmed subtype C lineage as well as Nef protein expression for a subset. The down-regulation of SERINC5 was measured by the co-transfection of the patient-derived Nef clones and a SERINC5 expression plasmid into a CEM-derived CD4 + T cell line, followed by detection of cell-surface SERINC5 using a fluorescently labelled antibody and flow cytometry. The ability of each Nef clone to down-regulate SERINC5 was expressed relative to the positive control (the highly functional SF2 subtype B Nef isolate, representing 100% activity) and a negative control (the defective G2A mutant of SF2, representing no activity) (Fig. 1a). The assay showed good reproducibility with a significant correlation between the duplicate measurements (Spearman’s correlation; r = 0.93 and *p* < 0.0001). Overall, Nef clones varied widely in SERINC5 down-regulation ability: the median SERINC5 down-regulation activity was 85% (interquartile range [IQR], 60–94%) (Fig. 1b).

The Nef clones were from three different cohorts, namely the HPP acute infection cohort (n = 32), Tshedimoso cohort (n = 27) and TRAPS cohort (n = 47) (Fig. 1b). While the sequences of the Nef clones from the different cohorts were previously shown to intermingle in a phylogenetic tree [15], there was a significant difference in the distribution of SERINC5 down-regulation values between the three cohorts (Kruskal–Wallis; *p* = 0.001). The highest median SERINC5 down-regulation activity was observed in the Tshedimoso cohort (95%; IQR, 74–97%) followed by 86% (IQR, 68–92%) in the TRAPS cohort and 66% (IQR, 46–91%) in the HPP acute infection cohort, where there was a significant difference specifically between the Tshedimoso and HPP acute infection cohorts (Dunn’s multiple comparisons test; *p* < 0.001). This cohort difference in SERINC5 down-regulation function warranted careful consideration of cohort effects in the models assessing the effect of SERINC5 down-regulation on viral load set point and rate of CD4 + T cell decline.

Previously, other Nef functions were measured for the same Nef clones [15, 16]. These included CD4 and HLA-I down-regulation, using flow cytometry-based methods similar to that for SERINC5 down-regulation [15]. Alteration of TCR signalling was also previously measured using a high throughput NFAT-based luciferase reporter T cell assay to measure the ability of each Nef clone to inhibit NFAT, a downstream molecule of TCR signalling, following TCR stimulation [16]. While there was significantly lower Nef-mediated alteration of TCR signalling activity in the Tshedimoso cohort compared to the HPP acute infection cohort, there was no difference in CD4/HLA-I down-regulation activities between the cohorts.

**Fig. 2:**
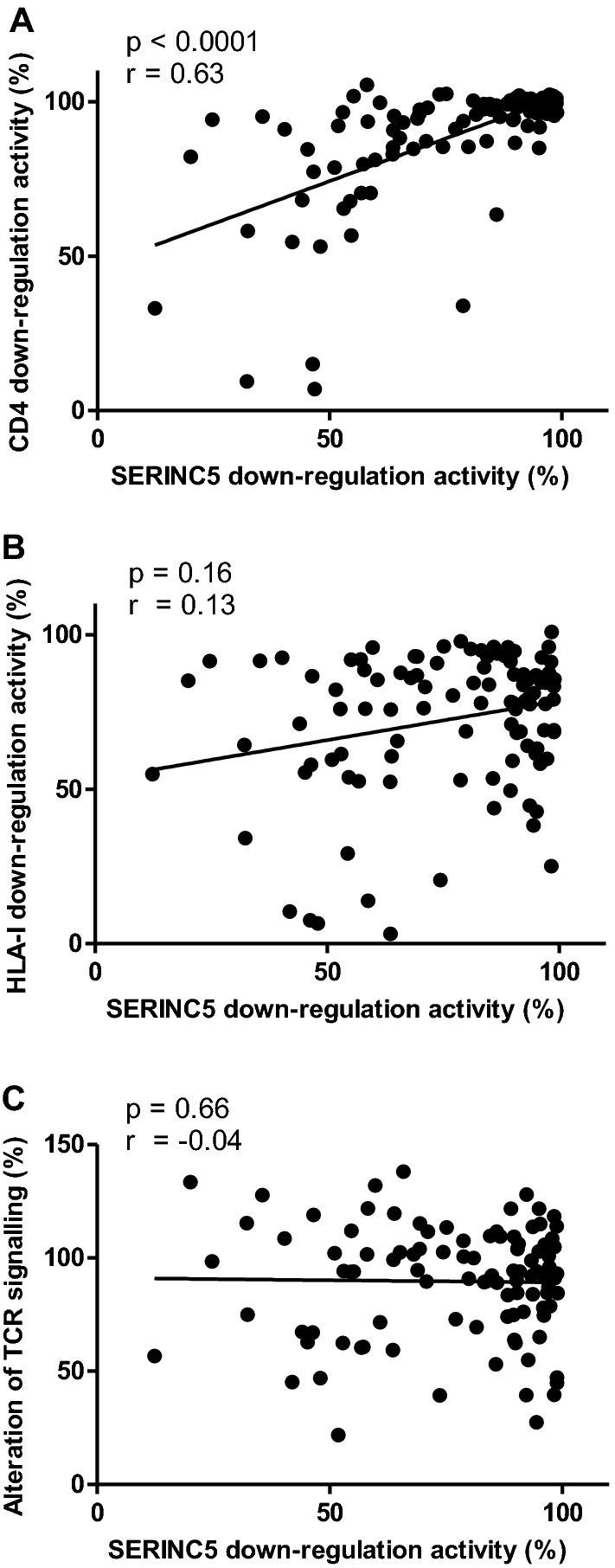
Comparison between SERINC5 down-regulation activity and the other Nef functions. **A**  The graph shows a statistically significant correlation (Spearman’s correlation) between SERINC5 down-regulation and CD4 down-regulation activities of patient-derived Nef clones, while graphs in panels **B** and **C** show no relationship between SERINC5 down-regulation activity and the activities of HLA-I down-regulation and alteration of TCR signalling. All Nef functions were expressed relative to that of SF2 Nef (100% activity)

All the Nef functional measurements for these Nef clones are available in Additional file 1. When investigating relationships between the different Nef functions, a statistically significant correlation between SERINC5 down-regulation activity and CD4 down-regulation activity (Spearman’s correlation; r = 0.63 and *p* < 0.0001) was observed (Fig. 2). On the other hand, there was no correlation observed between SERINC5 down-regulation activity and HLA-I down-regulation activity (Spearman’s correlation; r = 0.13 and *p* = 0.16) and neither between SERINC5 down-regulation activity and alteration of TCR signalling (Spearman’s correlation; r = − 0.04 and *p* = 0.66) (Fig. 2).

### SERINC5 down-regulation activity does not associate with markers of disease progression

In previous analyses of the same Nef clones, CD4 down-regulation function correlated positively with viral load set point and higher HLA-I down-regulation activity associated with a faster rate of CD4 + T cell decline, while the alteration of TCR signalling function did not correlate with either of these markers of disease progression [15, 16]. Nef-mediated SERINC5 down-regulation activity is responsible for enhancing virion infectivity, but it is not known to what extent SERINC5 down-regulation influences HIV-1 subtype C disease progression. To investigate this, the SERINC5 down-regulation activities of Nef clones derived from early infection were analysed together with subsequent viral load set point and the rate of CD4^+^ T cell decline using univariable and multivariable linear regression.

**Fig. 3:**
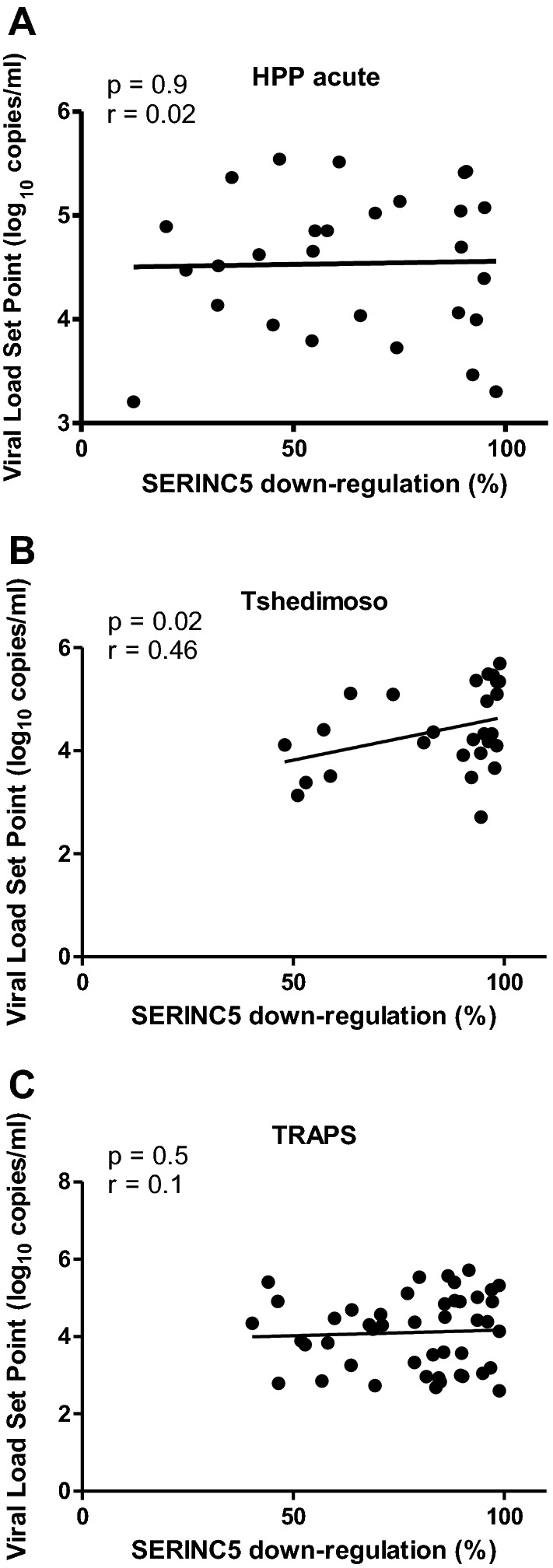
The relationship between SERINC5 down-regulation activity and viral load set point by cohort. The SERINC5 down-regulation activity was measured for Nef clones derived from three different cohorts, namely the HPP acute infection cohort (**A**), the Tshedimoso cohort (**B**) and the TRAPS cohort (**C**). SERINC5 down-regulation correlated significantly with viral load set point in the Tshedimoso cohort only (Spearman’s correlation). SERINC5 down-regulation activity was expressed relative to that of SF2 Nef (100% activity)

There was no significant effect of SERINC5 down-regulation on viral load set point in both the univariable and multivariable linear regression analyses (*p* = 0.59 and *p* = 0.28, respectively) (Table 1). Multivariable linear regression included cohort as a variable since there was a significant difference in SERINC5 down-regulation between cohorts. Interestingly, while there was no significant relationship between SERINC5 down-regulation and viral load set point overall, within the Tshedimoso cohort alone (the cohort with the highest SERINC5 down-regulation function) there was a significant positive correlation between SERINC5 down-regulation and viral load set point (Spearman’s correlation; r = 0.46 and *p* = 0.02) (Fig. 3).

**Table 1:** The association between SERINC5 down-regulation and viral load set point

Variable	Univariable (n = 101)			Variable	Multivariable (n = 101)		
	Coeff.	SE	*p* value		Coeff.	SE	*p* value
SERINC5^a^	0.22	0.4	0.59	SERINC5	0.46	0.42	0.28
				Cohort^b^: Tshedimoso	− 0.23	0.24	0.34
				TRAPS	− 0.5	0.21	0.02

Coeff., coefficient; SE, standard error

^a^SERINC5 down-regulation function is expressed relative to SF2 Nef (where SF2 function is 1 in the multivariable disease progression models as previously [15, 16])

^b^The HPP acute infection cohort is the reference group for cohorts

There was no significant effect of SERINC5 down-regulation on the rate of CD4^+^ T cell decline in univariable or multivariable analysis, which included cohort, follow-up time, baseline CD4 + T cell count and baseline viral load (*p* = 0.15 and *p* = 0.45, respectively) (Table 2).

**Table 2:** The association between SERINC5 down-regulation and rate of CD4 + T cell decline

Univariable (n = 101)				Multivariable (n = 101)			
Variable	Coeff.	SE	*p* value	Variable	Coeff.	SE	*p* value
SERINC5^a^	− 6.79	4.7	0.15	SERINC5	− 3.35	4.46	0.45
				Square-root baseline CD4^b^	− 0.99	0.24	< 0.0001
				Follow-up time^c^	0.01	0.002	< 0.0001
				Log baseline viral load^d^	− 1.03	0.9	0.25
				Cohort^e^: Tshedimoso	0.35	2.73	0.9
				TRAPS	− 2.59	2.44	0.29

CD4 + T cell decline values were limited to the range of − 50 to 50 cells/mm^3^ per month to exclude outliers

Coeff., coefficient; SE, standard error

^a^SERINC5 down-regulation function is expressed relative to SF2 Nef (where SF2 function is 1 in the multivariable disease progression models as previously [15, 16])

^b^CD4 + counts are expressed per one square root unit increase

^c^Follow-up time is expressed per day increment

^d^Viral load estimates are expressed per log10 increment

^e^The HPP acute infection cohort is the reference group for cohorts

In summary, there was no significant relationship between SERINC5 down-regulation and markers of disease progression overall.

### SERINC5 down-regulation contributes significantly to overall Nef function

**Fig. 4:**
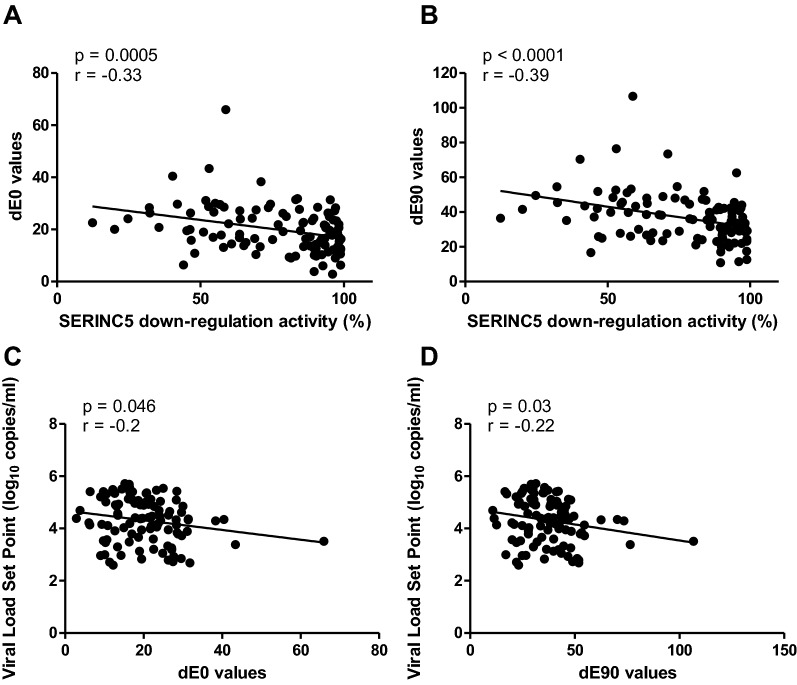
The relationship between dE0/dE90 values and SERINC5 down-regulation as well as viral load set point. SERINC5 down-regulation activity of patient-derived Nef clones (expressed relative to SF2 Nef, which represents 100% activity) correlated significantly (Spearman’s correlation) with dE0 values (**A**) and dE90 values (**B**), which are proxies for overall Nef function in vivo. dE0 values were derived from the Nef fitness landscape Ising model (only the consensus amino acid present at each residue was modelled explicitly) for each Nef clone, while dE90 values were derived from the Nef fitness landscape Potts model (each amino acid present at each residue was modelled explicitly) [17]. The dE0 values (**C**) and dE90 values (**D**) also correlated significantly with viral load set point (Spearman’s correlation)

An E value, which is a proxy for overall Nef function in vivo, has been predicted by computational modelling for each of the patient-derived Nef clones used in this study [17]. In past work, different Nef functional measurements were used as predictors of the E value with multiple linear regression to assess the contribution of each Nef function to overall Nef fitness [17]. In that study, CD4 down-regulation emerged as the strongest contributor of the Nef functions measured, however these measurements did not include SERINC5 down-regulation. Here, the contribution of SERINC5 down-regulation ability to overall Nef function was assessed using E values that were previously assigned to each of the clones and were derived from the Ising (dE0 values) and Potts models (dE90 values) [17] (dE0 and dE90 values for these clones are listed in Additional file 1). The Potts model accounts for the diversity of amino acids present at each residue. In contrast, in the Ising model, only the consensus amino acid present at each residue was modelled explicitly, and all other amino acids were treated as the same mutant type. A high dE0 or dE90 value is interpreted as corresponding to low in vivo Nef fitness (or a high fitness cost), while a low dE0 or dE90 value is interpreted as corresponding to high in vivo Nef fitness. The distribution of dE0 and dE90 values and their correlation with SERINC5 down-regulation values is shown in Fig. 4a, b (Spearman’s correlation; r = − 0.33 and *p* = 0.0005 for dE0 and r = − 0.39 and *p* < 0.0001 for dE90).

To allow for assessment of the relative contribution of each Nef function to dE0/dE90, Nef functions were standardised according to the means and the relationship between each standardised Nef function and dE0 was analysed using univariable and multivariable analysis (Table 3). In the univariable analysis, both CD4 down-regulation and SERINC5 down-regulation were significantly associated with dE0 with similar coefficients (− 2.22 and − 2.23) that were larger than those of the other Nef functions. A negative coefficient means that an increase in Nef function is associated with a decrease in dE0 value (i.e*.* an increase in in vivo Nef fitness). In multivariable analysis, however, these effects were not significant. We next explored whether the difference between the univariable and multivariable analysis was due to the possible impact of collinearity. This was considered due to the correlation between SERINC5 and CD4 down-regulation observed here, together with the correlation between HLA-I down-regulation and alteration of TCR signalling described previously [16]. This was, however, ruled out by assessing the variance inflation factors. A value greater than the threshold value of 5 would have suggested the influence of collinearity; however, all were below the threshold value of 5.

**Table 3:** The association between Nef functions and dE0 values

Nef function^a^	Univariable analysis			Multivariable analysis		
	Coeff.	SE	*p* value	Coeff.	SE	*p* value
CD4 down-regulation	− 2.22	1.11	0.046	− 0.31	1.76	0.86
HLA-I down-regulation	− 1.65	1.12	0.15	− 1.86	1.50	0.22
Alteration of TCR signalling	0.84	1.21	0.49	1.86	1.14	0.11
SERINC5 down-regulation	− 2.23	1.09	0.043	− 1.95	1.36	0.16

dE0 values were derived from the Nef fitness landscape Ising model (only the consensus amino acid present at each residue was modelled explicitly) for each Nef clone and serve as a proxy for in vivo Nef fitness [17]

Coeff., coefficient; SE, standard error

^a^Nef functions were standardised (by subtracting the mean and dividing by the standard deviation) so that coefficients were comparable

The relationship between each standardised Nef function and dE90 is presented in Table 4. In the univariable analysis, CD4 down-regulation and SERINC5 down-regulation were significantly associated with dE90 (*p* = 0.02 and *p* = 0.003, respectively). The greatest relative contribution to the overall Nef function was observed for SERINC5 down-regulation with a model coefficient of − 4.91 followed by CD4 down-regulation with a coefficient of − 3.64. However, in the multivariable analysis, only SERINC5 down-regulation remained statistically significant and the greatest driver of dE90, with a coefficient of − 5.32 (*p* = 0.003). This means that a one unit standardised increase in SERINC5 down-regulation function is associated with a 5.32 unit decrease in dE90.

**Table 4:** The association between Nef functions and dE90 values

Nef function^a^	Univariable analysis			Multivariable analysis		
	Coeff.	SE	*p* value	Coeff.	SE	*p* value
CD4 down-regulation	− 3.64	1.53	0.02	− 0.24	2.23	0.92
HLA-I down-regulation	− 2.10	1.59	0.19	0.19	1.91	0.92
Alteration of TCR signalling	0.99	1.70	0.56	1.26	1.46	0.39
SERINC5 down-regulation	− 4.91	1.60	0.003	− 5.32	1.73	0.003

dE90 values were derived from the Nef fitness landscape Potts model (each amino acid present at each residue was modelled explicitly) for each Nef clone and serve as a proxy for in vivo Nef fitness [17]

Coeff., coefficient; SE, standard error

^a^Nef functions were standardised (by subtracting the mean and dividing by the standard deviation) so that coefficients were comparable

Of interest, the dE0 and dE90 values were both correlated significantly with viral load set point (Spearman’s correlation; r = − 0.2 and *p* = 0.046, and r = − 0.22 and *p* = 0.03, respectively) (Fig. 4c, d), suggesting that the E value measure of overall Nef function has relevance for disease progression in vivo. However, neither dE0 (*p* = 0.85) or dE90 (*p* = 0.94) were significant predictors of the rate of CD4 + T cell decline in multivariable regression analysis that controlled for potential confounding factors (Tables 5, 6).

**Table 5:** The association between dE0 values and rate of CD4 + T cell decline

Variable	Multivariable, dE0 (n = 102)			Variable	Multivariable (n = 102)		
	Coeff.	SE	*p* value		Coeff.	SE	*p* value
dE0^a^	− 0.06	0.11	0.57	dE0	− 0.02	0.1	0.85
				Square-root baseline CD4^b^	− 0.99	0.24	< 0.0001
				Follow-up time^c^	0.01	0.002	< 0.0001
				Log baseline viral load^d^	− 1.25	0.9	0.17
				Cohort^e^: Tshedimoso	0.16	2.57	0.95
				TRAPS	− 2.89	2.38	0.23

CD4 + T cell decline values were limited to the range of − 50 to 50 cells/mm^3^ per month to exclude outliers

Coeff., coefficient; SE, standard error

^a^dE0 values were derived from the Nef fitness landscape Ising model (only the consensus amino acid present at each residue was modelled explicitly) for each Nef clone and serve as a proxy for in vivo Nef fitness [17]

^b^CD4 + counts are expressed per one square root unit increase

^c^Follow-up time is expressed per day increment

^d^ Viral load estimates are expressed per log10 increment

^e^The HPP acute infection cohort is the reference group for cohorts

**Table 6:** The association between dE90 values and rate of CD4 + T cell decline

Variable	Multivariable, dE0 (n = 102)			Variable	Multivariable (n = 102)		
	Coeff.	SE	*p* value		Coeff.	SE	*p* value
dE90^a^	− 0.05	0.07	0.51	dE90	− 0.004	0.06	0.94
				Square-root baseline CD4^b^	− 0.99	0.24	< 0.0001
				Follow-up time^c^	0.01	0.002	< 0.0001
				Log baseline viral load^d^	− 1.24	0.9	0.17
				Cohort^e^: Tshedimoso	0.15	2.57	0.95
				TRAPS	− 2.89	2.38	0.23

CD4 + T cell decline values were limited to the range of − 50 to 50 cells/mm^3^ per month to exclude outliers

Coeff., coefficient; SE, standard error

^a^dE90 values were derived from the Nef fitness landscape Potts model (each amino acid present at each residue was modelled explicitly) for each Nef clone and serve as a proxy for in vivo Nef fitness [17]

^b^CD4 + counts are expressed per one square root unit increase

^c^Follow-up time is expressed per day increment

^d^Viral load estimates are expressed per log10 increment

^e^The HPP acute infection cohort is the reference group for cohorts

In summary, the results suggest that CD4 down-regulation and SERINC5 down-regulation are the largest contributors of the Nef functions considered here to overall Nef function and that the contribution of SERINC5 down-regulation is the most significant. Results further suggest that overall Nef function affects viral load set point.

### Sequence determinants of Nef-mediated SERINC5 down-regulation activity

To identify Nef amino acids that either increase or decrease the ability of Nef to down-regulate SERINC5, a function-sequence analysis was performed using an online tool [18] that generates codon-by-codon Mann–Whitney U tests for every Nef amino acid variant present at least 5 times in the dataset.

We identified 15 amino acid variants at 11 different codons associated with altered SERINC5 down-regulation activity (Table 7). The most statistically significant association was observed at codon number 65, where Nef clones that encoded the consensus amino glutamic acid (n = 94) displayed lower SERINC5 down-regulation activity (median 81.3%) compared with clones that did not (n = 12; median 95.1%) (*p* = 0.0005). The amino acid variants that were associated with the largest alterations (by more than 30%) in SERINC5 down-regulation activity were at codons 10, 11, 38 and 173. Together these results suggest that natural polymorphisms in Nef can lead to both increased and decreased ability to down-regulate SERINC5.

**Table 7:** Nef amino acids significantly associated with altered SERINC5 down-regulation activity

Codon^a^	AA^b^	Cons^c^	Relative Nef function (%)^d^		Number of samples^e^		Impact^f^	*p* value	q value
			With AA	Without AA	With AA	Without AA			
10	K	I	57.6	88.3	6	86	− 31	0.008	0.3
10	I	I	88.6	60.9	78	14	28	0.01	0.3
11	V	V	89.5	58.0	79	13	32	0.003	0.3
38	D	D	89.5	65.9	77	29	24	0.005	0.3
38	E	D	55.1	86.7	13	93	− 32	0.01	0.3
51	T	N	92.7	79.9	25	81	13	0.01	0.3
65	E	E	81.3	95.1	94	12	− 14	0.0005	0.1
65	D	E	95.2	83.2	9	97	12	0.003	0.2
101	V	I	97.7	83.2	5	101	15	0.01	0.3
135	F	Y	59.8	85.9	13	93	− 26	0.03	0.4
173	T	M	54.6	85.9	5	101	− 31	0.02	0.3
176	T	E	58.8	86.7	9	97	− 28	0.02	0.4
188	H	S	94.4	83.2	6	100	12	0.01	0.3
191	H	R	91.0	81.3	16	90	10	0.03	0.4
191	R	R	81.3	91.0	88	18	− 10	0.03	0.4

Amino acids (AA) at n ≥ 5 in the dataset, *p* < 0.05, q ≤ 0.4

^a^Numbered according to HXB2

^b^The amino acids associated with increased or decreased SERINC5 down-regulation activity

^c^The consensus amino acid (Cons) at a particular codon from the reference 2004 consensus C Nef sequence from the Los Alamos HIV sequence database

^d^The median percentage SERINC5 down-regulation activity which was expressed relative to SF2 control (100% down-regulation activity) of the Nef clones with (+) and without (−) the amino acid

^e^The number of sequences with (+) and without (−) the amino acid

^f^The median Nef activity of the clones with the amino acid minus the median Nef activity of the clones without amino acid

**Fig. 5:**
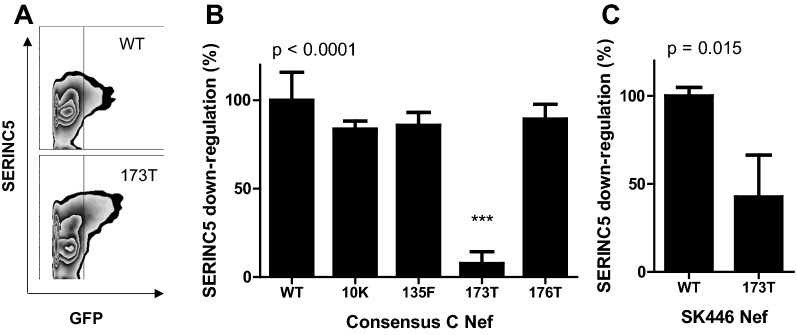
SERINC5 down-regulation activity of Nef mutants. **A** Flow cytometry plots of the consensus C Nef (WT) and the consensus C Nef 173T mutant (173T) are shown, with SERINC5 cell-surface expression on the y axis and the green fluorescent protein (GFP) expressing cells (representing cells transfected with Nef clones) on the x axis. The SERINC5 down-regulation activities of the consensus C Nef mutants (**B**) and SK446 Nef mutant (**C**) normalised to the respective WT proteins (representing 100% activity) are shown, where the data represents the means and standard deviations of three independent experiments. The SERINC5 down-regulation ability expressed relative to SF2 Nef was 80% and 91% for wild-type (WT) consensus C Nef and SK446 Nef, respectively. ANOVA with Tukey post-hoc tests was performed to assess which consensus C Nef mutants differed significantly from WT, and the Student’s T test was used to compare the down-regulation activity of the SK446 WT and SK446 173T mutant. The ANOVA/Student’s *T* test *p* values are shown and the Tukey post hoc test *p* value is indicated by asterisks (*** is *p* < 0.001)

To confirm the results of the codon-by-codon analysis, four amino acid variants (10K, 135F, 173T and 176T) were selected and introduced into a consensus C Nef by site-directed mutagenesis. While mutants 10K, 135F and 176T modestly reduced SERINC5 down-regulation activity (by 11–16%, *p* > 0.05), the 173T mutation markedly reduced SERINC5 down-regulation ability (reduced by 92% relative to wild-type; ANOVA with Tukey post-hoc test, *p* < 0.001) (Fig. 5). To confirm the deleterious effect of the 173T mutation on SERINC5 down-regulation, it was also introduced into a patient-derived Nef sequence (SK446). In the SK446 sequence background, 173T was similarly associated with a substantial reduction in Nef-mediated SERINC5 down-regulation activity (by 57%; Student’s T test, *p* = 0.015) (Fig. 5).

## Discussion

This study utilised Nef clones derived from early subtype C HIV-1 infection to investigate the contribution of SERINC5 down-regulation activity to HIV-1 subtype C disease progression as well as the relative contribution of this activity to overall Nef function, and to identify natural polymorphisms in subtype C Nef that influence this activity. Results suggest that, of the Nef functions studied here, SERINC5 down-regulation is the largest contributor to the dE0/dE90 values (representing overall Nef function), while CD4 down-regulation, but not HLA-I down-regulation or alteration of TCR signalling, is also a significant contributor. However, while dE0/dE90 values were positively associated with viral load set point, suggesting that they have relevance for disease progression in vivo, SERINC5 down-regulation activity did not associate with markers of disease progression.

The significant association between Nef fitness model dE0/dE90 values and the Nef activities of SERINC5 down-regulation and CD4 down-regulation, is consistent with studies showing the higher relative importance of Nef-mediated CD4 down-regulation and enhancement of virion infectivity for pathogenesis, when compared to activities relying on the SH3 domain of Nef [4]. Both dE0 values (from the Ising Nef fitness model) and dE90 values (from the Potts Nef fitness model) were considered as there are advantages and limitations to each model [17]. Since Nef is a highly mutable protein, the Potts model, which models each amino acid variant explicitly, is expected to be more reliable than the Ising model which uses a binary approximation (all mutant amino acids are denoted as one and the wild-type amino acid is denoted as zero). However, the Potts model advantage of residue-specific resolution may be offset by the disadvantage of introducing more noise into the model, and both models were found to perform similarly in predicting fitness costs in Nef [14].

While both CD4 and SERINC5 down-regulation were significantly associated with dE0/dE90 values (that correlated positively with viral load set point), only CD4 down-regulation was directly correlated with viral load set point in this dataset. The lack of correlation between SERINC5 down-regulation and viral load set point was unexpected given the association of this Nef functional measure with dE0/dE90 values (to a greater degree than CD4 down-regulation) and in light of previous studies showing effects of Nef-mediated CD4 down-regulation and/or enhancement of virion infectivity on clinical markers [3, 7, 19]. However, concerning the latter point, there may be other significant contributors to Nef-mediated enhancement of virion infectivity [20]. One interesting observation was the significant relationship between SERINC5 down-regulation and viral load set point in the Tshedimoso cohort from Botswana, but not in the other two cohorts from Durban. It may be possible that the significant difference in SERINC5 down-regulation function between the cohorts could have played a role in the lack of overall association between SERINC5 down-regulation and viral load set point. Previously we observed significantly lower Nef-mediated alteration of TCR signalling function in the Tshedimoso cohort compared to the HPP acute infection cohort [15], which was in line with previously reported lower Gag function in viruses derived from Botswana compared to those from Durban [21]. The opposite trend for Nef-mediated SERINC5 down-regulation is observed here, which suggests that a different factor is at play for SERINC5 down-regulation. Differences in SERINC5 expression level in the two populations could be explored as a possible factor contributing to the cohort difference observed in this study.

In an exploratory sequence-function analysis, several amino acid variants were found to associate with decreased (10K, 38E, 65E, 135F, 173T, 176T, and 191R) or increased (10I, 11V, 38D, 51T, 65D, 101V, 188H, and 191H) SERINC5 down-regulation activity. Some of these associations were consistent with those found in previous studies. For example, 65E was previously associated with lower SERINC5 down-regulation activity in subtype B Nef clones [6], and 51T was previously associated with higher SERINC5 down-regulation activity [6] as well as twofold higher infectivity [22] when compared with 51N in subtype B Nef sequences. Although the consensus amino acids at codons 10 and 11 differ between subtypes B and C, amino acid variations at codon 10 and 11 have been associated with altered virion infectivity [7] and SERINC5 down-regulation activity [15], respectively, in subtype B Nef clones. It was interesting that in the current study of subtype C Nef clones, the conservation of amino acids at codon 10 and 11 was associated with higher SERINC5 down-regulation activity, while conservation at amino acids 8–12 was previously associated with lower HLA-I down-regulation function in subtype C Nef clones [23]. On the other hand, the cytotoxic T cell escape mutation 135F was associated with lower SERINC5 down-regulation activity in our subtype C Nef clones, and was also previously shown to lower HLA-I down-regulation activity [24]. Somewhat consistent with the differing relationships between SERINC5 down-regulation and HLA-I down-regulation at different Nef codons observed here, was the overall lack of correlation between these two Nef functions in the current dataset, however a previous study in subtype B Nef clones did show some overlap between these two Nef activities albeit weaker than the relationship between SERINC5 and CD4 down-regulation [6]. 173T was one of the mutations associated with the largest reduction in SERINC5 down-regulation activity in the current study, which was confirmed by mutagenesis experiments, and 176T was also associated with lower activity. The effect of these residues on SERINC5 down-regulation is likely explained by their position adjacent to the E/DD (174–175) motif, which is crucial for Nef-AP-2 binding [25] and, consequently, SERINC5 down-regulation [10]. Several Nef motifs, including CAW (57–59), RR (105–106), LL(164–165), and E/DD (174–175), that are important for Nef-AP-2 interaction are key for both Nef-mediated SERINC5 down-regulation and CD4 down-regulation [10, 25]. The correlation between these two Nef functions in our dataset, as well as in previous studies [6], is consistent with the overlap in their genetic determinants.

An important limitation of the current study is that the role of Env in determining SERINC5 resistance independently of Nef was not taken into account [26]; i.e*.* it is possible that if Nef fails to downregulate SERINC5 well, the Env protein from the same virus could be highly resistant to SERINC5. It was also recently reported that the sensitivity of Env to SERINC5 antiviral activity is affected by CD4 expression in the producer cell, where CD4-Env interaction renders Env sensitive to SERINC5 [27]. It is therefore unclear whether CD4 down-regulation within a producer cell may affect sensitivity to SERINC5 antiviral activity (which could in turn affect the overall impact of SERINC5 down-regulation on viral load), although it is not likely since the CD4-Env interaction is hypothesised to occur co-translationally within the endoplasmic reticulum [27]. Further work is needed to better understand the interplay between SERINC5, Nef, Env and CD4. In addition, the level of expression of SERINC5 and Nef may affect the ability of Nef to down-regulate SERINC5 [11]. The assay used in the current study was previously optimised using differing amounts of SERINC5 to enhance detection of the effect of Nef on SERINC5 [14], however it may not be fully representative of the natural variation in SERINC5 expression. Lastly, it is important to note that other Nef functions that were not measured in this study, both known and unknown, could be just as important if not more important than the functions studied here.

In conclusion, this study suggested that Nef-mediated SERINC5 down-regulation function significantly contributes to overall Nef function and shed light on naturally-occurring Nef mutations that influence this Nef activity.

## Methods

### Patient-derived Nef clones

Nef-mediated SERINC5 down-regulation was evaluated for 106 Nef clones that were previously prepared by cloning patient Nef sequences into a pSELECT green fluorescent protein (GFP) reporter expression plasmid [15], and for which Nef activities of HLA-I and CD4 down-regulation, as well as alteration of TCR signalling, were previously measured [15, 16]. The 106 clones (one per individual) were derived from antiretroviral naïve patients with acute/early HIV-1 subtype C infection from three cohorts: the Tshedimoso study in Botswana [28], the HIV Pathogenesis Programme (HPP) Acute Infection Cohort in Durban, South Africa [29, 30], and the Tenofovir Gel Research for AIDS Prevention Science (TRAPS) Cohort in KwaZulu-Natal, South Africa [31]. The viral load and CD4^+^ T cell count information were available. The viral load set point was calculated as the average viral load from 3 to 12 months post-infection. Simple linear regression was used to compute the rate of CD4^+^ T cell decline (cells/mm^3^ per month) for every participant over the treatment-free follow-up period, where the rate of decline was defined as the estimated slope of the fitted regression line [30]. The Nef clone sequences are available under GenBank accession numbers KF208819, KF208821-3, KF208825-8, KF208831-4, KF208836, KF208838-9, KF208842-3, KF208845, KF208847-208853, KF208855, KF208857-208861, KF208863-5, KF208867, KF208870, KF208872-3, KF208878-9, KF208886, KF208889, KF208893-5, KM262907-262923, and KM262925-262968.

### SERINC5 down-regulation assay

The Nef-mediated down-regulation of SERINC5 was measured as previously described [6] with slight modifications. Briefly, one million HLA-A*02-expressing CEM-derived CD4 + T cells [23] were co-transfected with 2 µg of the Nef clone and 5 µg pSELECT-SERINC5-internal HA tag (iHA)-ΔGFP (sub-cloned from pBJ5-SERINC5(iHA) [8]) in 400 µl of Megacell (Sigma) via electroporation at 250 V and 950 μF using a Gene Pulser Xcell electroporator (BioRad). Following electroporation, 750 µl R10 medium (RPMI-1640 medium supplemented with 10% foetal bovine serum (Gibco), 10 mM HEPES buffer (Gibco), 2 mM L-glutamine (Sigma) and 50 U/ml penicillin–streptomycin (Gibco)) was added to the electroporated cells. The mixture was divided into 2 tubes (for duplicate staining) and then incubated at 37 ºC and 5% CO_2_ for 20 h to allow for the expression of Nef and SERINC5. Transfected cells in each tube were thereafter stained with 0.375 µg of a monoclonal Alexa Fluor anti-HA.11 Epitope Tag Antibody (BioLegend Way, California, USA). The level of SERINC5-HA expression was analyzed (through detection of the Alexa Fluor-labelled anti-HA antibody) in GFP-positive cells (representing Nef-transfected cells) using a FACS Calibur (BD Bioscience, San Jose, USA). The positive control was SF2 Nef and represented 100% down-regulation activity. A G2A clone (SF2 Nef harbouring a G2A mutation that completely abrogates Nef activity) was used as the negative control and represented 0% down-regulation activity. For each patient-derived Nef clone, the median fluorescence intensity (MFI) values of SERINC5 were normalised to that of the controls as follows: [(G2A Nef _MFI _− Nef clone _MFI_)/(G2A Nef _MFI _− SF2 Nef _MFI_)] × 100. Data from duplicate independent experiments, each with duplicate staining measurements, were averaged.

### Site-directed mutagenesis

Mutations of interest were introduced into the 2004 consensus C Nef sequence [32] as well as a patient-derived subtype C Nef sequence of high amino acid similarity (92.7%) to the consensus C sequence (SK446; GenBank accession KM263139). Prior to mutagenesis, the Nef sequences were cloned into a TOPO plasmid using the TOPO TA 3.1 cloning kit (Invitrogen, San Diego, USA). The Nef sequences in the TOPO plasmid were then mutated using the QuikChange II XL Site-Directed Mutagenesis kit (Agilent Technologies, Texas, USA) together with custom-designed mutagenic primers. Following confirmation of introduced mutations by sequencing using the ABI Prism Big Dye Terminator v3.1 Cycle Sequencing Kit (Applied Biosystems, Foster City, USA), the mutated Nef sequences were cloned into the pSELECT GFP reporter expression plasmid, as described previously [23], to allow for SERINC5 down-regulation analysis. Three independent SERINC5 down-regulation assays were performed for each Nef mutant and respective wild-type.

### Data analysis

Univariable and multivariable linear regression was used to assess the relationship between SERINC5 down-regulation and viral load set point or rate of CD4^+^ T cell decline. Multivariable analyses adjusted for cohort as a possible confounder. In addition, the CD4 + T cell decline analysis accounted for baseline CD4 + T cell count (square-root transformed), baseline viral load (log transformed), and follow-up time (in days). As previously [15, 16], extreme outliers of CD4 + T cell decline values were excluded (only values within the range of − 50 to 50 cells/mm^3^ per month were considered) to approximate a normal distribution, meet model assumptions and include > 95% of the dataset.

The relative contribution of each Nef function to overall Nef function (E value) was also assessed. An E value, which represents overall Nef fitness, has been predicted by computational modelling for each patient-derived Nef clone [17]. The dE0 value was derived from the Ising model where consensus versus mutant (all non-consensus amino acids are counted as the same mutant type) at each codon was modelled, and the dE90 value was derived from the Potts model where all amino acid variants at each codon are considered. In view of the skewness of distribution of dE0 and dE90 as well as the presence of outliers, analyses involving both dE0 values and dE90 values were performed using quantile (median) regression, which is more robust than standard linear regression. All Nef functions were standardised (by subtracting the mean and dividing by the standard deviation) so that coefficients were comparable. All regression analyses were performed using Stata 15.0 and *p* < 0.05 was considered significant.

Specific Nef amino acids (present at a frequency of n ≥ 5 in our dataset) significantly associated with increased or decreased SERINC5 down-regulation were assessed using codon-by-codon Mann–Whitney U tests, available online [18]. Multiple comparisons were addressed using q-values [33] and associations with *p* < 0.05 and q ≤ 0.4 were considered significant.

ANOVA with Tukey post-hoc tests (for more than 2 groups), or the Student’s T test (for 2 groups), was used to test for significant differences between the SERINC5 down-regulation activity of Nef mutants and that of their respective wild-type sequences. Analysis was performed using GraphPad Prism 5.01 and *p* < 0.05 was considered significant.

## Supplementary Information

**Additional file 1. In vitro Nef functional measurements and E values of patient-derived Nef clones.** Excel file showing in vitro Nef functional measurements (SERINC5 down-regulation, CD4 down-regulation, HLA-I down-regulation and alteration of TCR signalling) for Nef clones derived from individuals in early subtype C infection. Nef functions are expressed relative to SF2 Nef (where SF2 Nef function = 1). E values, which are a proxy for overall Nef function in vivo, have been predicted by computational modelling for each of these patient-derived Nef clones (17) and are shown alongside the in vitro Nef functional measurements. dE0 values were derived from the Nef fitness landscape Ising model (only the consensus amino acid present at each residue was modelled explicitly) for each Nef clone, while dE90 values were derived from the Nef fitness landscape Potts model (each amino acid present at each residue was modelled explicitly). Clinical measurements, including viral load set point (log10 copies/ml), rate of CD4+ T cell decline (cells/mm3 per month), baseline viral load (log10 copies/ml), baseline CD4+ T cell count (cells/mm3) and follow-up time (the number of days from first CD4+ T cell count to last CD4+ T cell count available) are also shown.

## Data Availability

All data generated or analysed during this study are included in this published article and its Additional file 1, with the exception of the Nef clone sequences which are available in GenBank (accession numbers provided in the article).
